# Antioxidative Properties and Inhibition of Key Enzymes Relevant to Type-2 Diabetes and Hypertension by Essential Oils from Black Pepper

**DOI:** 10.1155/2013/926047

**Published:** 2013-11-21

**Authors:** Ganiyu Oboh, Ayokunle O. Ademosun, Oluwatoyin V. Odubanjo, Ifeoluwa A. Akinbola

**Affiliations:** Functional Foods and Nutraceuticals Unit, Department of Biochemistry, Federal University of Technology, P M B. 704, Akure 340001, Nigeria

## Abstract

The antioxidant properties and effect of essential oil of black pepper (*Piper guineense*) seeds on **α**-amylase, **α**-glucosidase (key enzymes linked to type-2 diabetes), and angiotensin-I converting enzyme (ACE) (key enzyme linked to hypertension) were assessed. The essential oil was obtained by hydrodistillation and dried with anhydrous Na_2_SO_4_, and the phenolic content, radical [1,1-diphenyl-2 picrylhydrazyl (DPPH), 2,2′-azino-bis(3-ethylbenzthiazoline-6-sulphonic acid) (ABTS) and nitric oxide (NO)] scavenging abilities as well as the ferric reducing antioxidant property (FRAP) and Fe^2+^-chelating ability of the essential oil were investigated. Furthermore, the effect on **α**-amylase, **α**-glucosidase, and ACE enzyme activities was also investigated. The characterization of the constituents was done using GC. The essential oil scavenged DPPH∗, NO∗, and ABTS∗ and chelated Fe^2+^. **α**-Pinene, **β**-pinene, cis-ocimene, myrcene, allo-ocimene, and 1,8-cineole were among the constituents identified by GC. The essential oil inhibited **α**-amylase, **α**-glucosidase, and ACE enzyme activities in concentration-dependent manners, though exhibiting a stronger inhibition of **α**-glucosidase than **α**-amylase activities. Conclusively, the phenolic content, antioxidant activity, and inhibition of **α**-amylase, **α**-glucosidase, and angiotensin-1 converting enzyme activities by the essential oil extract of black pepper could be part of the mechanism by which the essential oil could manage and/or prevent type-2 diabetes and hypertension.

## 1. Introduction

Essential oils from aromatic spice plants have been shown to be good antioxidants using various antioxidant assay models [1–3] and the antioxidant activities have been linked to the phenolic contents in some of the oils [4, 5]. Furthermore, some medicinal properties of essential oils from aromatic spice plants have been established, especially essential oils from black pepper (*Piper guineense*) seeds which have been shown to have antimicrobial, antihypertensive, anticonvulsive, and sedative activities [5–7]. Black pepper (*Piper guineense*) is a spicy plant whose essential oils from the seed and leaves are being extracted and sold in commercial quantities in many countries [8]. Monoterpenes, benzoids, and sesquiterpenes have been identified among the volatile compounds of the black pepper [9]. 

In recent years, the management of type-2 diabetes and hypertension through natural sources has been done in two major ways: the scavenging of free radicals and inhibition of key enzymes involved in starch digestion (*α*-amylase and *α*-glucosidase) and high blood pressure (angiotensin-1 converting enzyme). Diabetes has been associated with an increased generation of free radicals and defective antioxidant defense systems [10, 11]. Furthermore, oxidative stress has been implicated in the diabetogenic process and in the physiological effects of diabetes [12, 13]. Therefore, antioxidant-rich foods have a good dietary intervention in the management of type-2 diabetes.

*α*-Amylase and *α*-glucosidase are two key enzymes that are therapeutic targets in the management of diabetes [14]. These two enzymes are involved in the breakdown of starch to glucose, thereby increasing the amount of glucose in the bloodstream. However, the inhibition of *α*-amylase and *α*-glucosidase activities delays the absorption of glucose and thereby moderates “postprandial blood glucose elevation” [15, 16]. Similarly, inhibition of angiotensin-1 converting enzyme (ACE) activity is a therapeutic approach to the management of hypertension as ACE converts angiotensin-I to angiotensin-II, which is a potent vasoconstrictor which leads to elevated blood pressure [17]. 

Essential oils from black pepper have been in use in folklore for the management of diabetes and hypertension. However, there is a dearth of information on the possible mechanism for the use of the essential oils for managing such degenerative conditions. This study, therefore sought to investigate the suitability of essential oils from black pepper seeds as a dietary means for the management of type-2 diabetes and hypertension by investigating the antioxidant properties of the oil and also its effect on *α*-amylase, *α*-glucosidase, and angiotensin-1 converting enzyme activities. 

## 2. Materials and Methods

### 2.1. Sample Collection

The Ashanti black peper (*Piper guineense*) was collected from the main market, Akure, south westren Nigeria (7.2500° N, 5.1950° E), over a period of seven days. The black pepper was ground to fine powder. The powder was stored in an air tight plastic container and placed at room temperature until it was used. 

### 2.2. Essential Oil Isolation

100 g of the ground Ashanti black peper was subjected to hydrodistillation for 3 h in an all glass Clevenger-type apparatus according to the method recommended by the European Pharmacopoeia [18]. The extracted oil sample was dried over anhydrous sodium sulphate and stored in sealed vials at 4°C for further analysis.

### 2.3. Reagents

All chemicals used in this study were of analytical grade and glass-distilled water was used.

### 2.4. Determination of Total Phenol Content

The total phenol content was determined according to the method of Singleton et al. [19]. Briefly, appropriate dilutions of the extract were oxidized with 2.5 mL 10% Folin-Ciocalteau's reagent (v/v) and neutralized by 2.0 mL of 7.5% sodium carbonate. The reaction mixture was incubated for 40 minute at 45°C and the absorbance was measured at 765 nm in the spectrophotometer. The total phenol content was subsequently calculated as gallic acid equivalent.

### 2.5. GC Analysis

The analytical GC was carried out by Hewlett Packard 5890 Gas Chromatograph (Hewlett-Packard Corp., Palo Alto, CA) equipped with flame ionization detectors (FID) with DB-5 column (30 m length, 0.25 mm column id., 0.25 *μ*m film thickness). The following conditions were applied: injection temperature: 290°C, injection volume: 1.0 *μ*L, injection mode: split (1 : 50), temperature program: 50°C for 4 min, rising at 3°C/min to 240°C, then rising at 15°C/min to 300°C, held at 300°C for 3 min, FID (290°C): H_2_, flow: 50 mL/min, and air flow: 400 mL/min.

### 2.6. 1,1-Diphenyl-2 Picrylhydrazyl (DPPH) Radical Scavenging Ability

The free radical scavenging ability of the oil against DPPH (1,1-diphenyl–2 picrylhydrazyl) free radical was evaluated as described by Gyamfi et al. [20]. Briefly, appropriate dilution of the extract (1 mL) was mixed with 1 mL, 0.4 mM methanolic solution containing DPPH radicals; the mixture was left in the dark for 30 min and the absorbance was taken at 516 nm. The DPPH free radical scavenging ability was subsequently calculated.

### 2.7. Fenton Reaction (Degradation of Deoxyribose)

The method of Halliwell and Gutteridge [21] was used to determine the ability of the extract to prevent Fe^2+^/H_2_O_2_ induced decomposition of deoxyribose. The extract 0–100 *μ*L was added to a reaction mixture containing 120 *μ*L of 20 mM deoxyribose, 400 *μ*L of 0.1 m phosphate buffer, and 40 *μ*L of 500 *μ*m of FeSO_4_, and the volume was made up to 800 *μ*L with distilled water. The reaction mixture was incubated at 37°C for 30 minutes and the reaction was then stopped by the addition of 0.5 mL of 28% trichloro acetic acid. This was followed by addition of 0.4 mL of 0.6% thiobarbituric acid solution. The tubes were subsequently incubated in boiling water for 20 minutes. The absorbance was measured at 532 nm in a spectrophotometer.

### 2.8. 2,2′-Azino-bis(3-ethylbenzthiazoline-6-sulphonic acid) (ABTS) Radical Scavenging Ability

The ABTS* scavenging ability of the essential oil was determined according to the method described by Re et al. [22]. The ABTS* was generated by the reaction of an (7 mmol/L) ABTS aqueous solution with K_2_S_2_O_8_ (2.45 mmol/L, final concentration) in the dark for 16 h and adjusting the Abs734 nm to 0.700 with ethanol. Samples of 0.2 mL of the extract were added to 2.0 mL ABTS* solution and the absorbance was measured at 734 nm after 15 mins. The trolox equivalent antioxidant capacity was subsequently calculated.

### 2.9. Nitric Oxide Radical Scavenging Assay

The scavenging effect of the extract on nitric oxide (NO^•^) radical was measured according to the method of Mercocci et al. [23]. Samples of 100–400 *μ*L of the oil extract were added in the test tubes to 1 mL of Sodium nitroprusside solution (25 mM) and tubes incubated at 37°C for 2 hours. An aliquot (0.5 mL) of the incubation was removed and diluted with 0.3 mL Griess reagent (1% sulphanilamide in 5% H_3_PO_4_ and 0.1% naphthlethylenediaminedihy drochloride). The absorbance of the chromophore formed was immediately read at 570 nm against distilled water as blank with catechin (50 *μ*g) used as standard. Results were expressed as percentage radical scavenging activity (RSA).

### 2.10. Determination of Reducing Property

The reducing property of the essential oil was determined by assessing the ability of the extract to reduce FeCl_3_ solution as described by Oyaizu [24]. 2.5 mL aliquot was mixed with 2.5 mL 200 mM sodium phosphate buffer (pH 6.6) and 2.5 mL 1% potassium ferricyanide. The mixture was incubated at 50°C for 20 min and then 2.5 mL of 10% trichloroacetic acid was added. This mixture was centrifuged at 650 g for 10 min. 5 mL of the supernatant was mixed with an equal volume of water and 1 mL of 0.1% ferric chloride. The absorbance was measured at 700 nm. The ferric reducing antioxidant property was subsequently calculated.

### 2.11. Fe^2+^ Chelation Assay

The Fe^2+^ chelating ability of the essential oil was determined using a modified method of Minotti and Aust [25] with a slight modification by Puntel et al. [26]. Freshly prepared 500 *μ*M FeSO_4_ (150 *μ*L) was added to a reaction mixture containing 168 *μ*L of 0.1 M Tris-HCl (pH 7.4), 218 *μ*L saline, and the extracts (0–25 *μ*L). The reaction mixture was incubated for 5 min, before the addition of 13 *μ*L of 0.25% 1, 10-phenanthroline (w/v). The absorbance was subsequently measured at 510 nm in a spectrophotometer. The Fe (II) chelating ability was subsequently calculated.

### 2.12. *α*-Amylase Inhibition Assay

The essential oil (500 *μ*L) and 500 *μ*L of 0.02 M sodium phosphate buffer (pH 6.9 with 0.006 M NaCl) containing hog pancreatic *α*-amylase (EC 3.2.1.1) (0.5 mg/mL) were incubated at 25°C for 10 minutes. Then, 500 *μ*L of 1% starch solution in 0.02 M sodium phosphate buffer (pH 6.9 with 0.006 M NaCl) was added to each tube. The reaction mixtures were incubated at 25°C for 10 minutes and stopped with 1.0 mL of dinitrosalicylic acid colour reagent. Thereafter, the mixture was incubated in a boiling water bath for 5 minutes and cooled to room temperature. The reaction mixture was then diluted by adding 10 mL of distilled water, and absorbance was measured at 540 nm [27].

### 2.13. *α*-Glucosidase Inhibition Assay

The essential oil (50 *μ*L) and 100 *μ*L of *α*-glucosidase solution (1.0 U/mL) in 0.1 M phosphate buffer (pH 6.9) were incubated at 25°C for 10 min. Then, 50 *μ*L of 5 mM p-nitrophenyl-*α*-D-glucopyranoside solution in 0.1 M phosphate buffer (pH 6.9) was added. The mixtures were incubated at 25°C for 5 min, before reading the absorbance at 405 nm in the spectrophotometer. The *α*-glucosidase inhibitory activity was expressed as percentage inhibition [28].

### 2.14. Angiotensin I Converting Enzyme (ACE) Inhibition Assay

The essential oil extract (50 *μ*L) and ACE solution (50 *μ*L, 4 mU) were incubated at 37°C for 15 min. The enzymatic reaction was initiated by adding 150 *μ*L of 8.33 mM of the substrate Bz-Gly-His-Leu in 125 mM Tris-HCl buffer (pH 8.3) to the mixture. After incubation for 30 min at 37°C, the reaction was arrested by adding 250 *μ*L of 1 M HCl. The Gly-His bond was then cleaved and the Bz-Gly produced by the reaction was extracted with 1.5 mL ethyl acetate. Thereafter the mixture was centrifuged to separate the ethyl acetate layer; then 1 mL of the ethyl acetate layer was transferred to a clean test tube and evaporated. The residue was redissolved in distilled water and its absorbance was measured at 228 nm. The ACE inhibitory activity was expressed as percentage inhibition [29].

### 2.15. Data Analysis

The results of three replicates were pooled and expressed as mean ± standard deviation (S.D.). Student's *t*-test, one-way analysis of variance (ANOVA), and least significance difference (LSD) were carried out [30]. Significance was accepted at *P* ≤ 0.05. EC_50_ was determined using linear regression analysis.

## 3. Results

**Figure 1:**
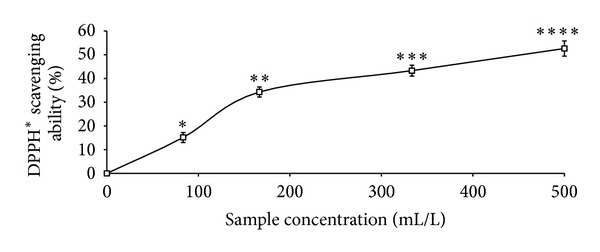
DPPH radical scavenging ability of essential oils from black pepper. Values represent means ± standard deviation of triplicate readings.

**Figure 2:**
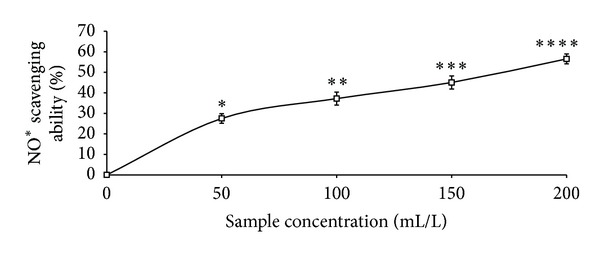
NO radical scavenging ability of essential oils from black pepper. Values represent means ± standard deviation of triplicate readings.

**Figure 3:**
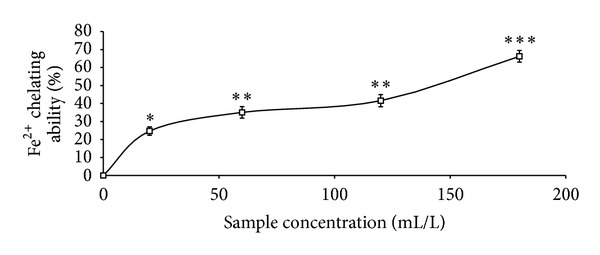
Fe^2+^chelating ability of essential oils from black pepper. Values represent means ± standard deviation of triplicate readings.

**Figure 4:**
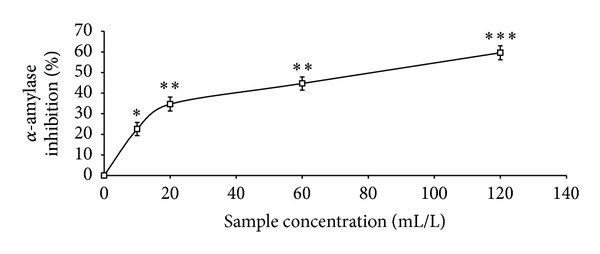
*α*-Amylase inhibitory activity of essential oils from black pepper. Values represent means ± standard deviation of triplicate readings.

**Figure 5:**
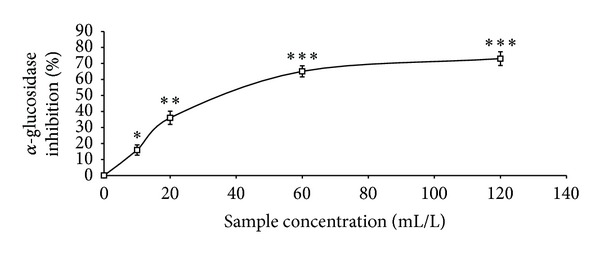
*α*-Glucosidase inhibitory activity of essential oils from black pepper. Values represent means ± standard deviation of triplicate readings.

**Figure 6:**
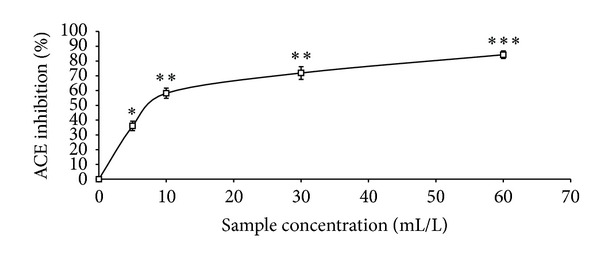
Angiotensin-I converting enzyme (ACE) inhibitory activity of essential oils from black pepper. Values represent means ± standard deviation of triplicate readings.

The total phenolic content reported as gallic acid equivalent, ABTS* scavenging ability reported as trolox equivalent, and ferric reducing antioxidant property reported as ascorbic acid equivalent as presented in Table 1 were 4.41 mg/100 g, 2.25 mmol./g, and 11.12 mg/100 g, respectively. The GC analysis as presented in Table 2 revealed the presence of *α*-pinene (13.63%), *β*-pinene (41.24%), cis-ocimene (3.63%), myrcene (4.37%), allo-ocimene (3.43%), pinene-2-ol (2.79%), *α*-thujene (2.98%), gamma terpinene (5.68%), and 1,8-cineole (17.22%). As shown in Figures 1, 2, and 3 and Table 3, the essential oil scavenged DPPH* and NO* and chelated Fe^2+^ in concentration-dependent manners, with EC_50_ values of 414.59 mL/L, 161.92 mL/L, and 130.21 mL/L, respectively. The effects of the essential oil on *α*-amylase, *α*-glucosidase, and angiotensin-I converting enzyme activities are presented in Figures 4, 5, and 6. The EC_50_ values as presented in Table 3 are 86.06 mL/L (*α*-amylase), 68.29 mL/L (*α*-glucosidase), and 28.99 mL/L (ACE).

## 4. Discussion

The link between free radical formation and the development and complications of diabetes has been well-established [31–33]. Furthermore, radical scavengers such as phenolics have been shown to be effective in preventing diabetes in animal models [31]. The radical scavenging abilities of the essential oil and its ferric reducing antioxidant property (FRAP) agree with previous findings by Politeo et al. [1]. Essential oils from Thymus species were also found by Amiri [5] to scavenge the radical. It is noteworthy, however, that the antiradical properties of the essential oils could be attributed to the presence of phenolic monoterpenes [34]. However, the individual and synergistic bioactivities of other compounds with strong antioxidant properties which were identified in the essential oil using GC would also have contributed to the observed antioxidant effects. The ferric reducing ability of the black pepper essential oil could be linked to the *α*-pinene and 1,8-cineole content, which have been shown to have reductive potentials [35]. Furthermore, the presence of functional groups such as “–OH”, “–O–”, and “–C = O–”, which are present in the volatile compounds of the oil in a favourable structure-function configuration, could contribute to the Fe^2+^ chelating ability [36, 37]. 

A modern therapeutic approach to the management of diabetes and its related complications is the inhibition of starch metabolizing enzymes such as *α*-amylase and *α*-glucosidase [38] as this will slow down the catabolism of starch into glucose and ultimately moderate the blood glucose level [39]. As presented in this study, black pepper essential oil extracts showed a concentration-dependent inhibition of *α*-amylase and *α*-glucosidase activities. Furthermore, the EC_50_ values revealed that the oil showed a stronger inhibition of *α*-glucosidase activity than *α*-amylase activity and this is therapeutically important in preventing some of the side effects associated with the use of synthetic *α*-amylase and *α*-glucosidase inhibitors [40]. Similarly, the inhibition of Angiotensin-1 converting enzyme (ACE) activity is a modern therapeutic approach in the management of hypertension which is one of the complications associated with type-2 diabetes [16]. The essential oil inhibited ACE activity *in vitro* in a concentration-dependent manner and therefore could have the ability to moderate the conversion of angiotensin-I to angiotensin-II which is a vasoconstrictor that has been implicated in the development of hypertension [41]. 

## 5. Conclusion

The antioxidant activity of essential oil from black pepper as well as its inhibition of *α*-amylase, *α*-glucosidase, and angiotensin-1 converting enzyme activities could be part of the mechanism by which the oil manages and/or prevents type-2 diabetes and hypertension. However, further *in vivo* experiments and clinical trials are recommended.

## Figures and Tables

**Table 1:** The total phenolic content reported as gallic acid equivalent, trolox equivalent antioxidant capacity and ferric reducing antioxidant property reported as ascorbic acid equivalent of essential oils from black pepper.

Parameter (unit)	Value
Total phenol (gallic acid equivalent) (mg/100 g)	4.41 ± 0.34
Trolox equivalent antioxidant capacity (mmol/g)	2.25 ± 0.32
Ferric reducing antioxidant property (ascorbic acid equivalent) (mg/100 g)	11.12 ± 0.83

Values represent means ± standard deviation of triplicate readings.

**Table 2:** Chemical composition of essential oils from black pepper.

Composant	RT	%
Camphene	4.738	0.26
Limonene	7.715	0.34
*α*-Pinene	9.428	13.63
*β*-Pinene	10.955	41.24
Benzyl alcohol	11.502	0.62
Cis-ocimene	12.526	3.63
Myrcene	13.042	4.37
Allo-ocimene	13.147	3.43
Pinene-2-ol	13.790	2.79
*α*-Thujene	14.176	2.98
Gamma terpinene	14.902	5.68
Neral	15.337	0.54
Geranial	15.405	0.43
Isoartemisia	16.364	0.26
1,8-Cineole	16.569	17.22
Linalool	17.665	0.87
Borneol	17.801	0.52
Terpinen-4-ol	18.544	0.29
*α*-Terpineol	18.710	0.28
Thymyl methyl ether	19.662	0.34
*α*-Copane	25.091	0.22

RT: retention time.

**Table 3:** EC_50_ of DPPH and NO radical scavenging abilities, Fe^2+^ chelating ability, and inhibition of *α*-amylase, *α*-glucosidase and angiotensin-1 converting enzyme activities by black pepper essential oil extracts.

Parameter	Value (mL/L)
DPPH*	414.59 ± 11.32
NO*	161.92 ± 14.26
Fe^2+^ chelation	130.21 ± 18.49
*α*-Amylase	86.06 ± 4.51
*α*-Glucosidase	68.29 ± 4.48
Angiotensin-1 converting enzyme	28.99 ± 2.68

Values represent means ± standard deviation of triplicate readings.
